# Multiparametric Cardiac Magnetic Resonance and Arrhythmias in Myocarditis

**DOI:** 10.3390/jcm12113754

**Published:** 2023-05-30

**Authors:** Patrycja S. Matusik, Tadeusz J. Popiela, Angeliki Darma, Enes E. Gul, Paweł T. Matusik

**Affiliations:** 1 Department of Diagnostic Imaging, University Hospital, 30-688 Kraków, Poland; 2 Chair of Radiology, Jagiellonian University Medical College, 31-501 Kraków, Poland; 3 Department of Cardiac Electrophysiology, Heart Centre of Leipzig, 04289 Leipzig, Germany; 4 Division of Cardiac Electrophysiology, Madinah Cardiac Centre, Madinah 42351, Saudi Arabia; 5 Department of Electrocardiology, Institute of Cardiology, Faculty of Medicine, Jagiellonian University Medical College, 31-202 Kraków, Poland; 6 Department of Electrocardiology, The John Paul II Hospital, 31-202 Kraków, Poland

Myocarditis is an inflammatory disease of the myocardium with a wide range of potential etiological factors, including a variety of infectious agents (mainly viral), systemic diseases, drugs, and toxins [1,2,3]. Moreover, vaccination with an mRNA vaccine against severe acute respiratory syndrome coronavirus 2 has been reported as a potential rare cause of mainly self-limiting clinically suspected myocarditis [3]. However, it should be mentioned that a temporal relationship between vaccination and myocarditis does not necessarily imply causation [3]. The diagnosis of myocarditis remains challenging owing to its heterogeneous presentation and rarity [2]. The gold standard for diagnostics and establishing the etiology of myocarditis is endomyocardial biopsy (EMB). However, EMB bears a risk of severe complications and may be associated with high rates of inconclusive or false negative results which may occur in up to 50% of cases, leading to underdiagnosis of the disease [4,5,6]. Biopsy-proven myocarditis is reported in 9–16% of adult patients with unexplained non-ischemic dilated cardiomyopathy [5]. The symptoms of myocarditis vary ranging from mild discomfort due to palpitations to more severe clinical manifestations such as acute-coronary-syndrome-like presentations and arrhythmias, including supraventricular, ventricular, and bradyarrhythmias, along with atrioventricular blocks of any degree [1]. Ventricular arrhythmias often occur in the case of cardiac sarcoidosis and giant cell myocarditis, with a prevalence of 55% and 29%, respectively [7]. In the mild course of myocarditis, arrhythmias are less frequent (overall prevalence <10%) and are more commonly of supraventricular origin [7].

Current European Society of Cardiology (ESC) [5,8] and American Heart Association (AHA) guidelines [6] consider cardiac magnetic resonance (CMR) a valuable method in patients with clinically suspected myocarditis. In this group of patients, CMR has the potential to provide both important diagnostic and prognostic information. Late gadolinium enhancement (LGE) is the strongest independent predictor of mortality in myocardial inflammation, with an over 8-fold increased risk for all-cause mortality and almost 13-fold increased risk for cardiac mortality [2]. CMR provides comprehensive information on cardiac structure and function and allows for multiparametric tissue characterization, including the presence, extent and pattern of myocardial edema, hyperemia, capillary leaking, focal scarring, and diffuse fibrosis. According to the updated version of the Lake Louise Criteria, a “2 out of 2” rule for the diagnosis of myocarditis is proposed, and myocarditis on CMR may be recognized when both one positive T1-based imaging criterion (increased regional or global native T1, as measured by T1 mapping or increased extracellular volume fraction or the presence of LGE in a non-ischemic distribution pattern) and one T2-based imaging criterion (increased regional or global native T2, as measured by T2 mapping or increased T2 signal on T2-weighted CMR images) are met [1]. Structural and functional myocardial abnormalities (ventricular dilatation, abnormal regional wall motion, and impairment of systolic or diastolic function) as well as pericardial abnormalities are used as supportive diagnostic criteria [1]. CMR has shown its usefulness in both acute and chronic myocarditis [9]. In the acute setting, LGE visualizes necrosis, while scarring may be observed at a chronic stage. However, the extent of myocardial edema may be less pronounced in patients with chronic myocarditis [9]. Therefore, it is favorable to perform CMR imaging in the early stage of the disease. Nevertheless, like any diagnostic method, CMR also has some limitations. For example, although there are no clear guidelines regarding detailed monitoring of patients with myocarditis, control-CMR three months post diagnosis may be considered [2]. Additionally, the usefulness of CMR may be limited in patients with chronic cardiomyopathy or with frequent supraventricular or ventricular arrhythmias [10].

In the *Journal of Clinical Medicine* Special Issue entitled “New Frontiers in Electrocardiography, Cardiac Arrhythmias, and Arrhythmogenic Disorders”, Ozierański et al., investigated 19,978 patients from the nationwide MYO-PL (the occurrence, trends, management and outcomes of patients with myocarditis in Poland) database who were hospitalized with suspected myocarditis [11]. In this population, the overall incidence rate of myocarditis ranged from 1.15 to 14 per 100,000 patients and was highest in patients aged 16–20 years. The five-year survival rates for patients with myocarditis were worse than in the general population and ranged from 56% to 99%. When assessing the occurrence of cardiac arrhythmias according to age (>20 vs. ≤20 years old), they observed that only tachycardia/palpitations and bradycardia were observed more commonly in younger patients than older ones (1.7% vs. 1.2% and 0.3% vs. 0.1%, respectively). Atrial fibrillation, paroxysmal tachycardia, and ventricular tachycardia were more frequently observed in patients >20 years old (4.8% vs. 0.2%, 1.1% vs. 0.5%, and 0.3% vs. 0.1%, respectively). In another paper, Ozierański et al., identified from the nationwide MYO-PL database 3659 patients aged 0–20 years hospitalized for myocarditis [12]. In the study, increasing incidence of myocarditis in this age group over the last ten years was observed [12]. Interestingly, they observed that higher rates of tachycardia/palpitations (3.3% vs. 1.2%, respectively) and paroxysmal tachycardia (1.3% and 0.3%, respectively) were observed in females when compared to males. These studies are particularly important in that they demonstrate that the application of diagnostic tests (e.g., C-reactive protein, troponins, brain natriuretic peptides, CMR, EMB, heart catheterization, and coronary angiography) is generally underutilized, and they emphasize that the management of myocarditis requires improvement. Importantly, the diagnostic use of CMR and EMB was particularly low. CMR was performed only in 3284 of the total 19,978 patients (16.4%) and EMB was performed only in 142 of the 19,978 patients (0.7%) [11]. In the second paper, the authors described no significant sex-related differences in the frequency of CMR and EMB performance, but overall, these procedures were also performed in a minority of patients (15.4% and 0.3%, respectively) [12].

Therefore, as indicated in the current ESC and AHA guidelines [5,6], we emphasize the role of CMR as a valuable technique in establishing the diagnosis of myocarditis. Novel CMR imaging and electrocardiographic modalities, such as texture analysis, myocardial strain analysis using feature tracking, wearables, and artificial intelligence techniques are under investigation and may help to optimize diagnostic and risk stratification in patients with clinically suspected myocarditis in the near future [13]. So far, the machine learning utilized in CMR imaging has been associated with reduced time required for image segmentation and analysis. Machine learning may also be valuable for the prediction of adverse cardiovascular events, improving traditional risk scores with novel CMR-derived parameters. However, it should be remembered that CMR cannot replace EMB in the diagnostic workup of myocarditis, especially in determining the etiology and potential referral for appropriate treatment (e.g., in the case of identification of a need for tailored immunosuppressive therapy which may reduce arrhythmia burden in EMB-proven non-infectious myocarditis [10]), highlighting the benefits of a more personalized approach to clinical management.

## Data Availability

Not applicable.
